# Crystal structure of octa-μ_3_-selenido-(*p*-toluene­sulfonato-κ*O*)penta­kis­(tri­ethyl­phosphane-κ*P*)-*octa­hedro*-hexa­rhenium(III) *p*-toluene­sulfonate di­chloro­methane disolvate

**DOI:** 10.1107/S2056989015014334

**Published:** 2015-08-06

**Authors:** Julia A. Edwards, Robert McDonald, Lisa F. Szczepura

**Affiliations:** a Department of Chemistry, Campus Box 4160, Illinois State University, Normal, IL 61790-4160, USA; b Department of Chemistry, University of Alberta, Edmonton, Alberta T6G 2G2, Canada

**Keywords:** crystal structure, rhenium complex, [Re_6_(μ_3_-Se)_8_]^2+^ cluster core, tosyl­ate

## Abstract

The title compound, [Re_6_Se_8_{O_3_SC_6_H_4_(CH_3_)}{P(C_2_H_5_)_3_}_5_](CH_3_C_6_H_4_SO_3_)·2CH_2_Cl_2_, contains the face-capped hexa­nuclear [Re_6_(μ_3_-Se)_8_]^2+^ cluster core. The [Re_6_Se_8_]^2+^ cluster core displays a non-crystallographic center of symmetry and is bonded through the Re^III^ atoms to five tri­ethyl­phosphane ligands and one *p*-toluene­sulfonate ligand. One *p*-toluene­sulfonate counter-ion and two di­chloro­methane solvent mol­ecules are also present in the asymmetric unit. One of the ethyl chains of one triethylphos­phane ligand and one of the CH_2_Cl_2_ solvent molecules are disordered over two sets of sites (occupancy ratios 0.65:0.35 and 0.5:0.5, respectively). The Re—O(sulfon­ate) bond length of 2.123 (5) Å is similar to other Re—O bond lengths of hexa­nuclear rhenium chalcogenide clusters containing other *O*-donor ligands such as dimethyl sulfoxide (DMSO), di­methyl­formamide (DMF) and hydroxide.

## Related literature   

Lindner & Grimmer (1971 ▸) reported the insertion of sulfur trioxide into the Re-alkyl bond of (*p*-tol­yl)Re(CO)_5_ to generate the first example of a rhenium complex to contain a tosyl­ate moiety. Later, Eremenko *et al.* (1993 ▸) determined the structure of [Re(P(O*i*Pr)_3_)_2_(CO)(NO)(OTs)_2_] (OTs^−^ = *p*-toluene­sulfonate anion) which represented the first structural report of a rhenium complex containing tosyl­ate ligands. In the synthesis of octa­hedral rhenium chalcogenide cluster complexes, the substitution of either halide or nitrile ligands has proven an effective means for generating a variety of new cluster complexes (Zheng & Holm, 1997 ▸; Knott *et al.*, 2013 ▸; Yoshimura *et al.*, 2000 ▸). Nitrile ligands are often considered weakly coordinating and substitution of alkyl and aryl nitrile ligands has often been used in single metal chemistry (Endres, 1987 ▸) and in the preparation of [Re_6_ *Q*_8_]^2+^ (*Q* = S or Se) cluster complexes (Zheng & Holm, 1997 ▸; Durham *et al.*, 2012 ▸). However, there have been reports of the hexa­nuclear rhenium selenide cluster core, [Re_6_Se_8_]^2+^, activating nitriles to undergo reactions other than substitution (Orto *et al.*, 2007 ▸; Szczepura *et al.*, 2007 ▸). While structural reports of rhenium chalcogenide clusters containing other oxygen donor ligands have been previously reported (Dorson *et al.*, 2009 ▸; Mironov *et al.*, 2011 ▸; Zheng & Holm, 1997 ▸; Zheng *et al.*, 1999 ▸), this report represents the first example of tosyl­ate coordination to a [Re_6_ *Q*_8_]^2+^ cluster core. The average Re—P bond length of the five terminal PEt_3_ ligands in the title compound [2.479 Å] is similar to that in other rhenium selenide clusters containing PEt_3_ ligands (Durham *et al.*, 2012 ▸, 2015 ▸; Knott *et al.*, 2013 ▸; Zheng & Holm, 1997 ▸; Zheng *et al.*, 1999 ▸).
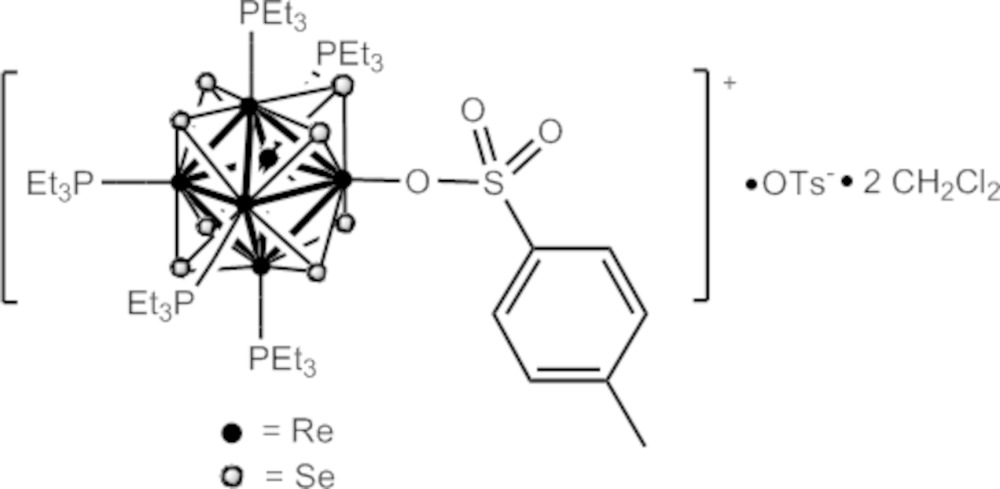


## Experimental   

### Crystal data  

[Re_6_Se_8_(C_7_H_7_O_3_S)(C_6_H_15_P)_5_](C_7_H_7_O_3_S)·2CH_2_Cl_2_
*M* *_r_* = 2851.85
Triclinic, 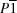

*a* = 11.7432 (10) Å
*b* = 16.6878 (14) Å
*c* = 18.9786 (16) Å
α = 93.4729 (11)°
β = 95.9862 (12)°
γ = 100.0851 (11)°
*V* = 3629.9 (5) Å^3^
*Z* = 2
Mo *K*α radiation
μ = 14.33 mm^−1^
*T* = 193 K
0.48 × 0.28 × 0.10 mm

### Data collection  

Bruker PLATFORM/SMART 1000 CCD area-detector diffractometer
Absorption correction: integration (*SADABS*; Sheldrick, 2008 ▸) *T*_min_ = 0.032, *T*_max_ = 0.255
31748 measured reflections
16405 independent reflections
11401 reflections with *I* > 2σ(*I*)
*R*_int_ = 0.031

### Refinement  

*R*[*F* ^2^ > 2σ(*F* ^2^)] = 0.036
*wR*(*F* ^2^) = 0.097
*S* = 1.02
16405 reflections
693 parameters
H-atom parameters constrained
Δρ_max_ = 2.67 e Å^−3^
Δρ_min_ = −1.26 e Å^−3^

### 

Data collection: *SMART* (Bruker, 2006 ▸); cell refinement: *SAINT* (Bruker, 2006 ▸); data reduction: *SAINT*; program(s) used to solve structure: *DIRDIF99* (Beurskens *et al.*, 2008 ▸); program(s) used to refine structure: *SHELXL2013* (Sheldrick, 2015 ▸); molecular graphics: *SHELXTL* (Sheldrick, 2008 ▸); software used to prepare material for publication: *SHELXTL*.

## Supplementary Material

Crystal structure: contains datablock(s) I, isu0804. DOI: 10.1107/S2056989015014334/pj2021sup1.cif

Structure factors: contains datablock(s) I. DOI: 10.1107/S2056989015014334/pj2021Isup2.hkl

**Fig. 1.**
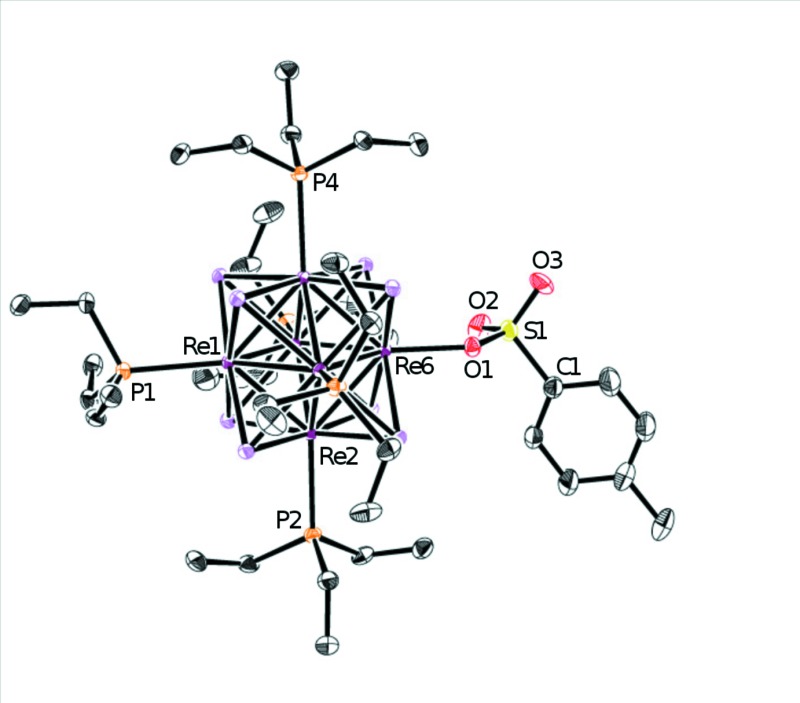
Perspective view of the [Re6Se8(PEt3)5(O3SC6H4Me)]+ ion showing the atom labelling scheme. Non-hydrogen atoms are represented by Gaussian ellipsoids at the 30% probability level. Hydrogen atoms omitted for clarity.

6 8 3 5 3 6 4 + . DOI: 10.1107/S2056989015014334/pj2021fig1.tif
Perspective view of the [Re_6_Se_8_(PEt_3_)_5_(O_3_SC_6_H_4_Me)]^+^ ion showing the atom labelling scheme. Non-hydrogen atoms are represented by Gaussian ellipsoids at the 30% probability level. Hydrogen atoms omitted for clarity.

CCDC reference: 1010097

Additional supporting information:  crystallographic information; 3D view; checkCIF report

## supplementary crystallographic information

### S1. Comment

In the synthesis of octahedral rhenium chalcogenide cluster complexes, the substitution of either halide or nitrile ligands has proven an effective means for generating a variety of new cluster complexes (Zheng & Holm, 1997, Knott *et al.*, 2013, Yoshimura *et al.*, 2000). Substitution of halide ligands can be facilitated by silver(I) salts, where the precipitation of AgX (*X* = halide) drives the substitution reaction (Zheng & Holm, 1999, Durham *et al.*, 2012). Nitrile ligands are often considered weakly coordinating and substitution of alkyl and aryl nitrile ligands has often been used in single metal chemistry (Endres, 1987) and in the preparation of [Re_6_Q_8_]^2+^ (Q = S or Se) cluster complexes (Zheng & Holm, 1997, Durham *et al.*, 2012). However, there have been reports of the hexanuclear rhenium selenide cluster core, [Re_6_Se_8_]^2+^, activating nitriles to undergo reactions other than substitution (Orto *et al.*, 2007, Szczepura *et al.*, 2007). To address this, we sought to incorporate a weakly coordinating ligand that would be readily substituted, but would not be susceptible to nucleophilic attack. The tosylate ligand was our ligand of choice and here we report the preparation, characterization and X-ray structural analysis of [Re_6_Se_8_(PEt_3_)_5_(OTs)](OTs)·2CH_2_Cl_2_. We recently substituted the tosylate ligand in this complex for a *N*-heterocyclic carbene which resulted in the formation of the first [*M*_6_Q_8_]^n+^ complex to contain a carbene ligand (Durham *et al.* 2015).

The structural data for the title complex, [Re_6_Se_8_(PEt_3_)_5_(OTs)](OTs)·2CH_2_Cl_2_, shows that the core contains bond lengths (Re–Re and Re–Se) and bond angles (Re–Re–Re, Re–Re–Se, Se–Re–Se, Re–Se–Re) that are consistent with other [Re_6_Se_8_]^2+^ based cluster complexes (Dorson *et al.*, 2009, Durham, *et al.*, 2012, 2015, Knott *et al.*, 2013, Mironov *et al.*, 2011, Zheng & Holm 1997, Zheng *et al.* 1999). The average Re—P bond distance of the five terminal PEt_3_ ligands is 2.4790 Å which also similarly compares with other rhenium selenide clusters containing PEt_3_ ligands (Durham, *et al.*, 2012, 2015, Knott *et al.*, 2013, Zheng & Holm, 1997, 1999). The Re6–O1 bond length of the coordinated tosylate is 2.123 (5) Å and the Re6–O1–S1 bond angle is 136.5 (5)*^o^*. The Re–O bond lengths of the coordinated tosylate ligands in [Re(P(O*i*Pr)_3_)_2_(CO)(NO)(OTs)_2_] were reported at 2.096 and 2.114 Å (Eremenko *et al.*, 1993). While there are no other structural reports of rhenium complexes containing tosylate ligands, there are a number of structural reports of rhenium trifluoromethanesulfonate (TfO^-^) complexes (Huertos *et al.*, 2010, Matano *et al.*, 1998, Tahmassebi *et al.*, 1997). Examining the structural data for these Re–OTf complexes, the Re–O bond lengths range from 2.10 - 2.53 Å and the Re–O–S bond angles typically fall between 124 - 139°; the data for the title complex fall well within the range observed for these relatively similar ligands. The Re6–O1 bond distance reported here also compares favorably with Re–O bond lengths of [Re_6_Se_8_]^2+^ clusters containing other O-donor ligands such as DMSO, DMF and hydroxide (Dorson *et al.* (2009), Mironov *et al.* (2011), Zheng & Holm (1997, Zheng *et al.* 1999)).

### S2. Experimental

The [Re_6_Se_8_(PEt_3_)_5_I]I complex was obtained according to a previously published procedure (Zheng *et al.*, 1997). The title complex is sensitive to water; therefore, this procedure was performed in an inert atmosphere glovebox. The starting cluster complex, [Re_6_Se_8_(PEt_3_)_5_I]I (199.9 mg, 0.0771 mmol) was dissolved in 20 ml of CH_2_Cl_2_ and placed in a round bottom flask. Separately, 61.2 mg (0.219 mmol) of AgOTs was dissolved in 5 ml of CH_2_Cl_2_ and transferred to the cluster solution. The flask was covered and allowed to stir overnight. The resulting slurry was filtered through Celite (to remove AgI) and the filtrate reduced to dryness. Reprecipitation using CH_2_Cl_2_ and Et_2_O allowed the product to be isolated (178 mg, 86% yield) and crystals were obtained *via* the vapor diffusion technique using CH_2_Cl_2_ and Et_2_O. ^1^H NMR (400 MHz, CDCl_3_, p.p.m.): 1.11 (45*H*, m, P(CH_2_C*H*_3_)), 2.05 (6*H*, m, P(C*H*_2_CH_3_)), 2.14 (24*H*, m P(C*H*_2_CH_3_)), 2.30 (3*H*, s, OSO_2_C_6_H_4_C*H*_3_ anion), 2.34 (3*H*, s, OSO_2_C_6_H_4_C*H*_3_ ligand), 7.08 (2*H*, d, OSO_2_C_6_*H*_4_CH_3_ anion), 7.13 (2*H*, d, OSO_2_C_6_*H*_4_CH_4_ ligand), 7.63 (2*H*, d, OSO_2_C_6_*H*_4_CH_3_ ligand), 7.88 (2*H*, d, OSO_2_C_6_*H*_4_CH_3_ anion). ^31^P {^1^H} NMR (162 MHz, CDCl_3_, p.p.m.): -26.54, -30.07. Anal. Calcd. for C_44_H_89_O_6_P_5_Re_6_S_2_Se_8_·2CH_2_Cl_2_: C, 19.37; H, 3.29; N, 0.00. Found: C, 19.26; H, 3.29; N, 0.13. ESI-MS(+): 2512.8 m/*z* [(*M*-OSO_2_C_6_H_4_CH_3_)^+^].

### S3. Refinement

Programs for diffractometer operation, data collection, data reduction and absorption correction were those supplied by Bruker. Hydrogen atoms were included as riding atoms and were placed in geometrically idealized positions with isotropic displacement parameters of 120% those of Ueq for their parent atoms. The methyl carbon of one of the phosphine ethyl groups was found to be disordered over two sites, for which the occupancy factors were adjusted in order to give the most satisfactory behavior of the displacement parameters and the bond lengths and angles, resulting in a final 65:35 distribution of occupancies. The chlorine atoms of one of the solvent CH2Cl2 molecules were also found to each be disordered over two positions, and adjustment of occupancies to give the best combination of displacement parameters and molecular geometry resulted in these atoms being assigned occupancies of 50%.

### Crystal data

**Table:**

[Re_6_Se_8_(C_7_H_7_O_3_S)(C_6_H_15_P)_5_](C_7_H_7_O_3_S)·2CH_2_Cl_2_	*Z* = 2
*M**_r_* = 2851.85	*F*(000) = 2628
Triclinic, *P*1	*D*_x_ = 2.609 Mg m^−^^3^
Hall symbol: -P 1	Mo *K*α radiation, λ = 0.71073 Å
*a* = 11.7432 (10) Å	Cell parameters from 7041 reflections
*b* = 16.6878 (14) Å	θ = 2.2–27.4°
*c* = 18.9786 (16) Å	µ = 14.33 mm^−^^1^
α = 93.4729 (11)°	*T* = 193 K
β = 95.9862 (12)°	Prism, orange
γ = 100.0851 (11)°	0.48 × 0.28 × 0.10 mm
*V* = 3629.9 (5) Å^3^	

### Data collection

**Table:**

Bruker PLATFORM/SMART 1000 CCD area-detector diffractometer	11401 reflections with *I* > 2σ(*I*)
Detector resolution: 8.192 pixels mm^-1^	*R*_int_ = 0.031
ω scans	θ_max_ = 27.5°, θ_min_ = 1.7°
Absorption correction: integration (*SADABS*; Sheldrick, 2008)	*h* = −15→15
*T*_min_ = 0.032, *T*_max_ = 0.255	*k* = −21→21
31748 measured reflections	*l* = −24→24
16405 independent reflections	

### Refinement

**Table:**

Refinement on *F*^2^	0 restraints
Least-squares matrix: full	Hydrogen site location: inferred from neighbouring sites
*R*[*F*^2^ > 2σ(*F*^2^)] = 0.036	H-atom parameters constrained
*wR*(*F*^2^) = 0.097	*w* = 1/[σ^2^(*F*_o_^2^) + (0.038*P*)^2^ + 7.5887*P*] where *P* = (*F*_o_^2^ + 2*F*_c_^2^)/3
*S* = 1.02	(Δ/σ)_max_ = 0.001
16405 reflections	Δρ_max_ = 2.67 e Å^−^^3^
693 parameters	Δρ_min_ = −1.26 e Å^−^^3^

### Special details

**Table:**

Geometry. All e.s.d.'s (except the e.s.d. in the dihedral angle between two l.s. planes) are estimated using the full covariance matrix. The cell e.s.d.'s are taken into account individually in the estimation of e.s.d.'s in distances, angles and torsion angles; correlations between e.s.d.'s in cell parameters are only used when they are defined by crystal symmetry. An approximate (isotropic) treatment of cell e.s.d.'s is used for estimating e.s.d.'s involving l.s. planes.

### Fractional atomic coordinates and isotropic or equivalent isotropic displacement parameters (Å^2^)

**Table:**

	*x*	*y*	*z*	*U*_iso_*/*U*_eq_	Occ. (<1)
Re1	0.12366 (2)	0.35432 (2)	0.22926 (2)	0.02007 (6)	
Re2	0.16617 (2)	0.29786 (2)	0.10389 (2)	0.02159 (7)	
Re3	0.13895 (2)	0.19879 (2)	0.20606 (2)	0.02062 (7)	
Re4	0.30352 (2)	0.29772 (2)	0.29017 (2)	0.02004 (6)	
Re5	0.33294 (2)	0.39696 (2)	0.18802 (2)	0.02136 (7)	
Re6	0.34623 (2)	0.24274 (2)	0.16411 (2)	0.02221 (7)	
Se1	−0.02863 (6)	0.25580 (4)	0.14801 (3)	0.02465 (15)	
Se2	0.10186 (6)	0.25694 (4)	0.32525 (3)	0.02334 (14)	
Se3	0.28647 (6)	0.44606 (4)	0.30833 (3)	0.02378 (14)	
Se4	0.15816 (6)	0.44579 (4)	0.12967 (4)	0.02754 (16)	
Se5	0.18447 (6)	0.15003 (4)	0.08552 (3)	0.02631 (15)	
Se6	0.31453 (6)	0.14981 (4)	0.26240 (4)	0.02578 (15)	
Se7	0.49890 (6)	0.33842 (4)	0.24662 (4)	0.02539 (15)	
Se8	0.36656 (6)	0.34011 (4)	0.06760 (4)	0.02695 (15)	
S1	0.58942 (18)	0.19272 (12)	0.10790 (11)	0.0404 (5)	
P1	−0.02451 (16)	0.42936 (10)	0.27116 (10)	0.0267 (4)	
P2	0.06449 (18)	0.29468 (11)	−0.01759 (9)	0.0289 (4)	
P3	0.01218 (17)	0.06768 (10)	0.22232 (10)	0.0277 (4)	
P4	0.39486 (17)	0.29590 (11)	0.41376 (9)	0.0266 (4)	
P5	0.47147 (19)	0.52475 (11)	0.17694 (10)	0.0357 (5)	
O1	0.4663 (4)	0.1748 (3)	0.1248 (3)	0.0323 (11)	
O2	0.6306 (5)	0.2788 (3)	0.1027 (3)	0.0562 (17)	
O3	0.6607 (5)	0.1522 (4)	0.1537 (3)	0.0618 (18)	
C1	0.5763 (8)	0.1441 (5)	0.0219 (5)	0.046 (2)	
C2	0.6345 (11)	0.0810 (7)	0.0078 (6)	0.083 (4)	
H2	0.6805	0.0625	0.0456	0.100*	
C3	0.6272 (11)	0.0436 (7)	−0.0609 (6)	0.077 (3)	
H3	0.6694	0.0011	−0.0697	0.092*	
C4	0.5604 (10)	0.0679 (6)	−0.1147 (6)	0.064 (3)	
C5	0.4992 (13)	0.1288 (8)	−0.1005 (6)	0.108 (5)	
H5	0.4500	0.1456	−0.1375	0.130*	
C6	0.5091 (13)	0.1662 (9)	−0.0313 (6)	0.122 (6)	
H6	0.4667	0.2085	−0.0224	0.146*	
C7	0.5535 (11)	0.0278 (7)	−0.1896 (6)	0.083 (4)	
H7A	0.6287	0.0130	−0.1970	0.100*	
H7B	0.5344	0.0662	−0.2242	0.100*	
H7C	0.4929	−0.0214	−0.1958	0.100*	
C11	−0.0042 (7)	0.4563 (4)	0.3667 (4)	0.0351 (17)	
H11A	−0.0040	0.4056	0.3910	0.042*	
H11B	0.0737	0.4910	0.3791	0.042*	
C12	−0.0937 (8)	0.5010 (5)	0.3965 (4)	0.046 (2)	
H12A	−0.0747	0.5115	0.4482	0.055*	
H12B	−0.1715	0.4672	0.3857	0.055*	
H12C	−0.0925	0.5530	0.3749	0.055*	
C13	−0.1760 (7)	0.3747 (4)	0.2540 (4)	0.0373 (18)	
H13A	−0.2280	0.4115	0.2693	0.045*	
H13B	−0.1959	0.3610	0.2022	0.045*	
C14	−0.1991 (7)	0.2963 (4)	0.2920 (4)	0.0391 (18)	
H14A	−0.2825	0.2732	0.2845	0.047*	
H14B	−0.1742	0.3086	0.3430	0.047*	
H14C	−0.1552	0.2568	0.2729	0.047*	
C15	−0.0325 (7)	0.5260 (4)	0.2323 (4)	0.0377 (18)	
H15A	−0.0461	0.5153	0.1800	0.045*	
H15B	−0.1008	0.5465	0.2478	0.045*	
C16	0.0724 (8)	0.5918 (4)	0.2507 (4)	0.045 (2)	
H16A	0.0568	0.6425	0.2316	0.053*	
H16B	0.1388	0.5755	0.2301	0.053*	
H16C	0.0905	0.6004	0.3025	0.053*	
C21	−0.0445 (7)	0.2022 (5)	−0.0443 (4)	0.0394 (19)	
H21A	−0.0043	0.1548	−0.0431	0.047*	
H21B	−0.0999	0.1961	−0.0082	0.047*	
C22	−0.1147 (8)	0.1979 (5)	−0.1174 (4)	0.049 (2)	
H22A	−0.1685	0.1454	−0.1261	0.059*	
H22B	−0.0616	0.2030	−0.1540	0.059*	
H22C	−0.1590	0.2425	−0.1188	0.059*	
C23	0.1580 (7)	0.3016 (5)	−0.0883 (4)	0.0392 (19)	
H23A	0.1103	0.3071	−0.1333	0.047*	
H23B	0.2176	0.3520	−0.0780	0.047*	
C24	0.2195 (8)	0.2302 (5)	−0.0994 (4)	0.046 (2)	
H24A	0.2675	0.2394	−0.1384	0.055*	
H24B	0.1617	0.1800	−0.1111	0.055*	
H24C	0.2693	0.2250	−0.0557	0.055*	
C25	−0.0151 (7)	0.3782 (5)	−0.0342 (4)	0.0410 (19)	
H25A	0.0408	0.4305	−0.0252	0.049*	
H25B	−0.0466	0.3727	−0.0850	0.049*	
C26	−0.1155 (8)	0.3824 (5)	0.0105 (5)	0.053 (2)	
H26A	−0.1515	0.4293	−0.0013	0.064*	
H26B	−0.0856	0.3884	0.0611	0.064*	
H26C	−0.1737	0.3321	0.0004	0.064*	
C31	−0.0210 (8)	−0.0054 (4)	0.1442 (4)	0.042 (2)	
H31A	0.0532	−0.0123	0.1263	0.051*	
H31B	−0.0572	−0.0588	0.1592	0.051*	
C32	−0.0993 (9)	0.0163 (5)	0.0844 (4)	0.058 (3)	
H32A	−0.1149	−0.0281	0.0464	0.069*	
H32B	−0.0618	0.0666	0.0660	0.069*	
H32C	−0.1728	0.0244	0.1013	0.069*	
C33	−0.1291 (7)	0.0800 (5)	0.2480 (4)	0.0395 (19)	
H33A	−0.1165	0.1133	0.2941	0.047*	
H33B	−0.1665	0.1110	0.2123	0.047*	
C34	−0.2127 (8)	0.0017 (5)	0.2552 (5)	0.055 (2)	
H34A	−0.2861	0.0147	0.2687	0.066*	
H34B	−0.1784	−0.0288	0.2918	0.066*	
H34C	−0.2277	−0.0315	0.2097	0.066*	
C35	0.0707 (7)	0.0060 (4)	0.2885 (4)	0.0348 (17)	
H35A	0.0161	−0.0467	0.2865	0.042*	
H35B	0.1452	−0.0060	0.2745	0.042*	
C36	0.0925 (8)	0.0420 (5)	0.3650 (4)	0.046 (2)	
H36A	0.1228	0.0031	0.3953	0.055*	
H36B	0.0193	0.0531	0.3805	0.055*	
H36C	0.1494	0.0930	0.3687	0.055*	
C41	0.5134 (7)	0.3820 (5)	0.4418 (4)	0.0407 (19)	
H41A	0.5739	0.3808	0.4093	0.049*	
H41B	0.4827	0.4330	0.4357	0.049*	
C42	0.5712 (8)	0.3856 (5)	0.5176 (4)	0.050 (2)	
H42A	0.6305	0.4353	0.5275	0.060*	
H42B	0.6080	0.3377	0.5236	0.060*	
H42C	0.5124	0.3861	0.5506	0.060*	
C43	0.4584 (7)	0.2066 (4)	0.4322 (4)	0.0375 (19)	
H43A	0.4822	0.2090	0.4840	0.045*	
H43B	0.3972	0.1575	0.4197	0.045*	
C44	0.5638 (8)	0.1958 (5)	0.3935 (5)	0.049 (2)	
H44A	0.5945	0.1483	0.4098	0.059*	
H44B	0.6244	0.2448	0.4039	0.059*	
H44C	0.5399	0.1874	0.3422	0.059*	
C45	0.2999 (7)	0.2951 (5)	0.4839 (4)	0.0393 (19)	
H45A	0.2359	0.2473	0.4733	0.047*	
H45B	0.3451	0.2874	0.5292	0.047*	
C46	0.2465 (8)	0.3717 (5)	0.4943 (4)	0.045 (2)	
H46A	0.1957	0.3650	0.5322	0.054*	
H46B	0.2008	0.3799	0.4500	0.054*	
H46C	0.3087	0.4192	0.5075	0.054*	
C51	0.6216 (9)	0.5128 (6)	0.1577 (5)	0.064 (3)	
H51A	0.6737	0.5665	0.1691	0.076*	
H51B	0.6495	0.4747	0.1907	0.076*	
C52	0.6356 (9)	0.4832 (6)	0.0850 (5)	0.070 (3)	
H52A	0.7187	0.4872	0.0802	0.085*	
H52B	0.6011	0.5165	0.0509	0.085*	
H52C	0.5964	0.4261	0.0756	0.085*	
C53	0.5125 (10)	0.5942 (5)	0.2573 (5)	0.068 (3)	
H53A	0.5558	0.6466	0.2444	0.081*	
H53B	0.4406	0.6054	0.2758	0.081*	
C54	0.5877 (9)	0.5626 (6)	0.3171 (5)	0.067 (3)	
H54A	0.6022	0.6021	0.3588	0.080*	
H54B	0.6620	0.5556	0.3008	0.080*	
H54C	0.5466	0.5101	0.3297	0.080*	
C55	0.4257 (12)	0.5886 (6)	0.1134 (6)	0.089 (4)	
H55A	0.4962	0.6185	0.0958	0.107*	0.65
H55B	0.3803	0.5530	0.0727	0.107*	0.65
H55C	0.4270	0.5578	0.0673	0.107*	0.35
H55D	0.3422	0.5864	0.1187	0.107*	0.35
C56A	0.3580 (17)	0.6468 (11)	0.1335 (10)	0.085 (5)*	0.65
H56A	0.3389	0.6772	0.0926	0.102*	0.65
H56B	0.4024	0.6848	0.1721	0.102*	0.65
H56C	0.2859	0.6187	0.1495	0.102*	0.65
C56B	0.466 (3)	0.672 (2)	0.1006 (19)	0.094 (11)*	0.35
H56D	0.4193	0.6859	0.0591	0.112*	0.35
H56E	0.5481	0.6785	0.0915	0.112*	0.35
H56F	0.4598	0.7074	0.1423	0.112*	0.35
S2	0.1203 (2)	0.25430 (12)	0.69762 (10)	0.0397 (5)	
O4	0.2051 (6)	0.3129 (3)	0.7426 (3)	0.0593 (18)	
O5	0.0437 (5)	0.2020 (3)	0.7371 (3)	0.0578 (17)	
O6	0.0570 (8)	0.2922 (4)	0.6441 (4)	0.085 (3)	
C61	0.1949 (7)	0.1916 (4)	0.6514 (4)	0.0346 (17)	
C62	0.1339 (8)	0.1340 (5)	0.5971 (4)	0.0409 (19)	
H62	0.0527	0.1310	0.5847	0.049*	
C63	0.1924 (9)	0.0821 (5)	0.5618 (5)	0.053 (2)	
H63	0.1498	0.0432	0.5257	0.064*	
C64	0.3105 (9)	0.0847 (5)	0.5772 (5)	0.050 (2)	
C65	0.3699 (8)	0.1410 (5)	0.6309 (5)	0.055 (2)	
H65	0.4511	0.1438	0.6432	0.066*	
C66	0.3122 (8)	0.1937 (5)	0.6671 (5)	0.046 (2)	
H66	0.3551	0.2321	0.7035	0.055*	
C67	0.3712 (11)	0.0272 (5)	0.5381 (6)	0.081 (4)	
H67A	0.3421	0.0223	0.4874	0.097*	
H67B	0.3559	−0.0266	0.5569	0.097*	
H67C	0.4552	0.0483	0.5443	0.097*	
Cl1S	0.1511 (3)	0.67463 (18)	0.49345 (15)	0.0790 (8)	
Cl2S	0.1300 (3)	0.8404 (2)	0.53900 (19)	0.0944 (10)	
C1S	0.0595 (10)	0.7443 (7)	0.5080 (6)	0.079 (3)	
H1SA	0.0109	0.7478	0.4629	0.094*	
H1SB	0.0065	0.7231	0.5427	0.094*	
Cl3S	0.2826 (7)	0.8779 (5)	0.3829 (4)	0.105 (3)*	0.5
Cl4S	0.3255 (6)	0.7327 (4)	0.3274 (3)	0.0828 (17)*	0.5
Cl5S	0.3045 (6)	0.9045 (4)	0.3569 (4)	0.0836 (19)*	0.5
Cl6S	0.3205 (7)	0.7571 (5)	0.2778 (4)	0.109 (2)*	0.5
C2S	0.2295 (12)	0.8018 (8)	0.3232 (8)	0.109 (5)	
H2SA	0.2221	0.8218	0.2752	0.131*	0.5
H2SB	0.1515	0.7749	0.3332	0.131*	0.5
H2SC	0.2069	0.7694	0.3632	0.131*	0.5
H2SD	0.1580	0.8048	0.2916	0.131*	0.5

### Atomic displacement parameters (Å^2^)

**Table:**

	*U*^11^	*U*^22^	*U*^33^	*U*^12^	*U*^13^	*U*^23^
Re1	0.02175 (14)	0.01753 (12)	0.02081 (13)	0.00464 (10)	0.00118 (11)	−0.00026 (9)
Re2	0.02488 (15)	0.01949 (13)	0.01971 (13)	0.00389 (11)	0.00088 (11)	−0.00048 (10)
Re3	0.02332 (15)	0.01668 (12)	0.02117 (13)	0.00340 (10)	0.00110 (11)	−0.00064 (9)
Re4	0.02163 (14)	0.01778 (13)	0.02046 (13)	0.00447 (10)	0.00114 (11)	−0.00061 (9)
Re5	0.02378 (15)	0.01844 (13)	0.02146 (14)	0.00346 (11)	0.00243 (11)	−0.00009 (10)
Re6	0.02424 (15)	0.01994 (13)	0.02252 (14)	0.00545 (11)	0.00282 (11)	−0.00207 (10)
Se1	0.0241 (4)	0.0240 (3)	0.0248 (3)	0.0040 (3)	−0.0001 (3)	−0.0005 (3)
Se2	0.0249 (4)	0.0217 (3)	0.0231 (3)	0.0036 (3)	0.0033 (3)	0.0007 (2)
Se3	0.0261 (4)	0.0196 (3)	0.0246 (3)	0.0035 (3)	0.0019 (3)	−0.0027 (2)
Se4	0.0326 (4)	0.0225 (3)	0.0278 (4)	0.0069 (3)	0.0012 (3)	0.0029 (3)
Se5	0.0310 (4)	0.0220 (3)	0.0246 (3)	0.0045 (3)	0.0014 (3)	−0.0044 (3)
Se6	0.0291 (4)	0.0202 (3)	0.0285 (4)	0.0072 (3)	0.0008 (3)	0.0011 (3)
Se7	0.0235 (4)	0.0252 (3)	0.0271 (4)	0.0050 (3)	0.0021 (3)	−0.0010 (3)
Se8	0.0302 (4)	0.0275 (3)	0.0227 (3)	0.0037 (3)	0.0047 (3)	0.0000 (3)
S1	0.0315 (11)	0.0419 (11)	0.0480 (12)	0.0094 (9)	0.0045 (9)	−0.0030 (9)
P1	0.0279 (10)	0.0244 (9)	0.0291 (9)	0.0091 (8)	0.0029 (8)	0.0005 (7)
P2	0.0353 (11)	0.0283 (9)	0.0227 (9)	0.0068 (8)	0.0001 (8)	0.0017 (7)
P3	0.0296 (10)	0.0206 (8)	0.0299 (10)	−0.0010 (8)	−0.0001 (8)	0.0008 (7)
P4	0.0301 (10)	0.0268 (9)	0.0226 (9)	0.0065 (8)	0.0000 (8)	0.0013 (7)
P5	0.0435 (13)	0.0264 (10)	0.0342 (11)	−0.0022 (9)	0.0063 (9)	0.0013 (8)
O1	0.030 (3)	0.025 (2)	0.041 (3)	0.005 (2)	0.004 (2)	−0.002 (2)
O2	0.046 (4)	0.040 (3)	0.074 (4)	−0.012 (3)	0.015 (3)	−0.012 (3)
O3	0.037 (4)	0.090 (5)	0.062 (4)	0.030 (4)	−0.006 (3)	0.007 (4)
C1	0.040 (5)	0.046 (5)	0.050 (5)	0.010 (4)	0.007 (4)	−0.004 (4)
C2	0.103 (10)	0.093 (9)	0.072 (8)	0.061 (8)	0.018 (7)	0.009 (7)
C3	0.094 (9)	0.070 (7)	0.078 (8)	0.043 (7)	0.020 (7)	−0.003 (6)
C4	0.078 (8)	0.052 (6)	0.064 (7)	0.014 (6)	0.027 (6)	−0.008 (5)
C5	0.147 (13)	0.145 (12)	0.056 (7)	0.106 (11)	0.000 (8)	−0.019 (7)
C6	0.184 (16)	0.161 (13)	0.048 (6)	0.143 (13)	−0.023 (8)	−0.027 (7)
C7	0.102 (10)	0.074 (7)	0.078 (8)	0.023 (7)	0.036 (7)	−0.022 (6)
C11	0.036 (5)	0.034 (4)	0.038 (4)	0.014 (3)	0.008 (4)	−0.002 (3)
C12	0.062 (6)	0.045 (5)	0.034 (4)	0.021 (4)	0.006 (4)	−0.001 (4)
C13	0.042 (5)	0.037 (4)	0.034 (4)	0.017 (4)	−0.008 (4)	0.000 (3)
C14	0.030 (4)	0.042 (4)	0.044 (5)	0.002 (4)	0.008 (4)	0.005 (4)
C15	0.045 (5)	0.029 (4)	0.044 (5)	0.018 (4)	0.008 (4)	0.009 (3)
C16	0.056 (6)	0.028 (4)	0.051 (5)	0.011 (4)	0.013 (4)	0.004 (4)
C21	0.040 (5)	0.040 (4)	0.036 (4)	0.006 (4)	0.000 (4)	0.000 (3)
C22	0.044 (5)	0.056 (5)	0.043 (5)	0.009 (4)	−0.006 (4)	−0.005 (4)
C23	0.051 (5)	0.040 (4)	0.024 (4)	−0.001 (4)	0.006 (4)	0.002 (3)
C24	0.050 (6)	0.053 (5)	0.036 (5)	0.009 (4)	0.011 (4)	−0.002 (4)
C25	0.051 (5)	0.040 (4)	0.032 (4)	0.013 (4)	−0.005 (4)	0.007 (3)
C26	0.058 (6)	0.051 (5)	0.053 (5)	0.030 (5)	−0.010 (5)	0.004 (4)
C31	0.054 (6)	0.025 (4)	0.043 (5)	−0.002 (4)	0.002 (4)	−0.005 (3)
C32	0.067 (7)	0.055 (6)	0.040 (5)	−0.007 (5)	−0.013 (5)	0.000 (4)
C33	0.035 (5)	0.038 (4)	0.043 (5)	−0.001 (4)	0.002 (4)	0.007 (3)
C34	0.042 (5)	0.046 (5)	0.076 (7)	−0.001 (4)	0.004 (5)	0.018 (5)
C35	0.035 (4)	0.022 (3)	0.046 (5)	0.002 (3)	0.004 (4)	0.009 (3)
C36	0.058 (6)	0.037 (4)	0.040 (5)	0.005 (4)	−0.005 (4)	0.012 (4)
C41	0.041 (5)	0.041 (4)	0.039 (4)	0.008 (4)	−0.001 (4)	0.001 (4)
C42	0.050 (6)	0.045 (5)	0.047 (5)	0.003 (4)	−0.013 (4)	−0.012 (4)
C43	0.050 (5)	0.033 (4)	0.034 (4)	0.020 (4)	0.000 (4)	0.008 (3)
C44	0.048 (5)	0.047 (5)	0.057 (5)	0.023 (4)	0.002 (4)	0.003 (4)
C45	0.039 (5)	0.049 (5)	0.031 (4)	0.012 (4)	0.004 (4)	0.004 (3)
C46	0.047 (5)	0.056 (5)	0.034 (4)	0.014 (4)	0.008 (4)	−0.008 (4)
C51	0.060 (7)	0.059 (6)	0.067 (7)	−0.009 (5)	0.010 (5)	0.020 (5)
C52	0.071 (8)	0.053 (6)	0.082 (8)	−0.010 (5)	0.029 (6)	−0.010 (5)
C53	0.090 (8)	0.044 (5)	0.051 (6)	−0.022 (5)	−0.012 (6)	−0.005 (4)
C54	0.070 (7)	0.066 (6)	0.048 (6)	−0.028 (5)	0.004 (5)	−0.005 (5)
C55	0.122 (11)	0.049 (6)	0.087 (8)	0.004 (7)	−0.023 (8)	0.025 (6)
S2	0.0549 (14)	0.0320 (10)	0.0315 (10)	0.0047 (9)	0.0068 (10)	0.0020 (8)
O4	0.069 (5)	0.044 (3)	0.058 (4)	−0.013 (3)	0.025 (3)	−0.013 (3)
O5	0.053 (4)	0.046 (3)	0.074 (4)	−0.002 (3)	0.031 (4)	−0.008 (3)
O6	0.134 (7)	0.088 (5)	0.056 (4)	0.083 (5)	0.015 (4)	0.001 (4)
C61	0.040 (5)	0.026 (4)	0.036 (4)	0.003 (3)	0.001 (4)	0.009 (3)
C62	0.049 (5)	0.040 (4)	0.033 (4)	0.008 (4)	0.004 (4)	0.000 (3)
C63	0.080 (8)	0.038 (5)	0.042 (5)	0.010 (5)	0.013 (5)	−0.004 (4)
C64	0.062 (7)	0.031 (4)	0.057 (6)	0.006 (4)	0.017 (5)	0.004 (4)
C65	0.043 (5)	0.039 (5)	0.088 (7)	0.012 (4)	0.013 (5)	0.021 (5)
C66	0.045 (5)	0.034 (4)	0.054 (5)	−0.001 (4)	0.001 (4)	−0.003 (4)
C67	0.118 (10)	0.045 (6)	0.101 (9)	0.042 (6)	0.058 (8)	0.019 (6)
Cl1S	0.085 (2)	0.0841 (19)	0.0724 (18)	0.0330 (17)	0.0007 (16)	0.0012 (15)
Cl2S	0.084 (2)	0.080 (2)	0.114 (3)	0.0180 (18)	−0.008 (2)	−0.0070 (19)
C1S	0.067 (8)	0.101 (9)	0.068 (7)	0.022 (7)	0.000 (6)	0.005 (7)
C2S	0.077 (10)	0.109 (11)	0.135 (12)	0.022 (8)	−0.012 (9)	−0.020 (9)

### Geometric parameters (Å, º)

**Table:**

Re1—P1	2.4818 (18)	P2—C25	1.833 (7)
Re1—Se2	2.5171 (7)	P3—C33	1.819 (8)
Re1—Se3	2.5199 (7)	P3—C31	1.825 (7)
Re1—Se4	2.5218 (7)	P3—C35	1.825 (7)
Re1—Se1	2.5232 (7)	P4—C43	1.816 (7)
Re1—Re2	2.6341 (4)	P4—C45	1.822 (7)
Re1—Re3	2.6464 (4)	P4—C41	1.826 (8)
Re1—Re4	2.6480 (4)	P5—C55	1.760 (10)
Re1—Re5	2.6486 (4)	P5—C53	1.827 (9)
Re2—P2	2.4765 (18)	P5—C51	1.880 (10)
Re2—Se4	2.5074 (7)	C1—C6	1.327 (13)
Re2—Se8	2.5150 (8)	C1—C2	1.380 (12)
Re2—Se1	2.5168 (7)	C2—C3	1.399 (14)
Re2—Se5	2.5201 (7)	C3—C4	1.347 (14)
Re2—Re3	2.6320 (4)	C4—C5	1.372 (13)
Re2—Re6	2.6321 (4)	C4—C7	1.523 (13)
Re2—Re5	2.6482 (4)	C5—C6	1.403 (14)
Re3—P3	2.4771 (17)	C11—C12	1.526 (10)
Re3—Se6	2.5097 (7)	C13—C14	1.529 (10)
Re3—Se1	2.5144 (7)	C15—C16	1.495 (11)
Re3—Se2	2.5170 (7)	C21—C22	1.528 (10)
Re3—Se5	2.5227 (7)	C23—C24	1.512 (10)
Re3—Re4	2.6322 (4)	C25—C26	1.532 (11)
Re3—Re6	2.6356 (4)	C31—C32	1.487 (11)
Re4—P4	2.4796 (18)	C33—C34	1.513 (10)
Re4—Se7	2.5152 (7)	C35—C36	1.515 (10)
Re4—Se2	2.5167 (7)	C41—C42	1.520 (10)
Re4—Se6	2.5209 (7)	C43—C44	1.535 (11)
Re4—Se3	2.5242 (7)	C45—C46	1.530 (10)
Re4—Re5	2.6360 (4)	C51—C52	1.471 (13)
Re4—Re6	2.6387 (4)	C53—C54	1.540 (13)
Re5—P5	2.4798 (19)	C55—C56A	1.418 (19)
Re5—Se4	2.5148 (8)	C55—C56B	1.43 (3)
Re5—Se7	2.5171 (7)	S2—O4	1.435 (6)
Re5—Se8	2.5171 (7)	S2—O6	1.438 (7)
Re5—Se3	2.5243 (7)	S2—O5	1.441 (6)
Re5—Re6	2.6206 (4)	S2—C61	1.733 (8)
Re6—O1	2.123 (5)	C61—C66	1.373 (11)
Re6—Se6	2.5131 (7)	C61—C62	1.409 (10)
Re6—Se7	2.5149 (7)	C62—C63	1.380 (11)
Re6—Se5	2.5154 (7)	C63—C64	1.380 (13)
Re6—Se8	2.5236 (7)	C64—C65	1.384 (12)
S1—O3	1.426 (6)	C64—C67	1.497 (11)
S1—O2	1.445 (6)	C65—C66	1.391 (11)
S1—O1	1.497 (5)	Cl1S—C1S	1.746 (11)
S1—C1	1.759 (9)	Cl2S—C1S	1.709 (12)
P1—C11	1.821 (7)	Cl3S—C2S	1.639 (14)
P1—C15	1.826 (7)	Cl4S—C2S	1.747 (14)
P1—C13	1.838 (8)	Cl5S—C2S	1.829 (14)
P2—C23	1.818 (7)	Cl6S—C2S	1.684 (14)
P2—C21	1.832 (8)		
			
P1—Re1—Se2	92.64 (4)	Se7—Re5—Re4	58.377 (17)
P1—Re1—Se3	92.22 (5)	Se8—Re5—Re4	119.023 (19)
Se2—Re1—Se3	89.78 (2)	Se3—Re5—Re4	58.524 (17)
P1—Re1—Se4	92.08 (4)	Re6—Re5—Re4	60.260 (10)
Se2—Re1—Se4	175.28 (2)	P5—Re5—Re2	136.47 (5)
Se3—Re1—Se4	89.95 (2)	Se4—Re5—Re2	58.040 (18)
P1—Re1—Se1	91.99 (5)	Se7—Re5—Re2	118.484 (19)
Se2—Re1—Se1	89.41 (2)	Se8—Re5—Re2	58.208 (19)
Se3—Re1—Se1	175.74 (2)	Se3—Re5—Re2	117.88 (2)
Se4—Re1—Se1	90.52 (2)	Re6—Re5—Re2	59.939 (10)
P1—Re1—Re2	134.53 (4)	Re4—Re5—Re2	89.800 (12)
Se2—Re1—Re2	118.069 (18)	P5—Re5—Re1	137.68 (5)
Se3—Re1—Re2	118.568 (19)	Se4—Re5—Re1	58.401 (18)
Se4—Re1—Re2	58.149 (17)	Se7—Re5—Re1	118.507 (19)
Se1—Re1—Re2	58.373 (17)	Se8—Re5—Re1	117.85 (2)
P1—Re1—Re3	135.44 (4)	Se3—Re5—Re1	58.245 (17)
Se2—Re1—Re3	58.284 (16)	Re6—Re5—Re1	90.185 (11)
Se3—Re1—Re3	118.027 (19)	Re4—Re5—Re1	60.143 (10)
Se4—Re1—Re3	117.904 (19)	Re2—Re5—Re1	59.644 (11)
Se1—Re1—Re3	58.147 (17)	O1—Re6—Se6	91.02 (13)
Re2—Re1—Re3	59.793 (9)	O1—Re6—Se7	94.31 (13)
P1—Re1—Re4	135.62 (4)	Se6—Re6—Se7	89.57 (2)
Se2—Re1—Re4	58.255 (17)	O1—Re6—Se5	88.99 (13)
Se3—Re1—Re4	58.415 (17)	Se6—Re6—Se5	89.52 (2)
Se4—Re1—Re4	117.82 (2)	Se7—Re6—Se5	176.59 (2)
Se1—Re1—Re4	117.766 (18)	O1—Re6—Se8	92.75 (13)
Re2—Re1—Re4	89.846 (12)	Se6—Re6—Se8	176.16 (2)
Re3—Re1—Re4	59.625 (10)	Se7—Re6—Se8	90.87 (2)
P1—Re1—Re5	135.03 (4)	Se5—Re6—Se8	89.82 (2)
Se2—Re1—Re5	117.935 (19)	O1—Re6—Re5	137.09 (13)
Se3—Re1—Re5	58.408 (17)	Se6—Re6—Re5	118.684 (18)
Se4—Re1—Re5	58.147 (18)	Se7—Re6—Re5	58.655 (17)
Se1—Re1—Re5	118.510 (19)	Se5—Re6—Re5	119.115 (19)
Re2—Re1—Re5	60.172 (10)	Se8—Re6—Re5	58.553 (17)
Re3—Re1—Re5	89.532 (11)	O1—Re6—Re2	133.56 (13)
Re4—Re1—Re5	59.693 (11)	Se6—Re6—Re2	118.24 (2)
P2—Re2—Se4	92.27 (4)	Se7—Re6—Re2	119.176 (19)
P2—Re2—Se8	94.73 (5)	Se5—Re6—Re2	58.571 (17)
Se4—Re2—Se8	88.80 (2)	Se8—Re6—Re2	58.348 (18)
P2—Re2—Se1	88.81 (5)	Re5—Re6—Re2	60.551 (10)
Se4—Re2—Se1	91.00 (2)	O1—Re6—Re3	132.52 (13)
Se8—Re2—Se1	176.46 (2)	Se6—Re6—Re3	58.289 (18)
P2—Re2—Se5	91.59 (4)	Se7—Re6—Re3	118.238 (19)
Se4—Re2—Se5	176.02 (3)	Se5—Re6—Re3	58.592 (18)
Se8—Re2—Se5	89.92 (2)	Se8—Re6—Re3	118.29 (2)
Se1—Re2—Se5	90.05 (2)	Re5—Re6—Re3	90.372 (12)
P2—Re2—Re3	132.58 (5)	Re2—Re6—Re3	59.953 (11)
Se4—Re2—Re3	118.978 (19)	O1—Re6—Re4	136.22 (13)
Se8—Re2—Re3	118.744 (19)	Se6—Re6—Re4	58.533 (17)
Se1—Re2—Re3	58.412 (17)	Se7—Re6—Re4	58.365 (17)
Se5—Re2—Re3	58.587 (17)	Se5—Re6—Re4	118.46 (2)
P2—Re2—Re6	136.88 (5)	Se8—Re6—Re4	118.677 (19)
Se4—Re2—Re6	117.82 (2)	Re5—Re6—Re4	60.158 (9)
Se8—Re2—Re6	58.667 (18)	Re2—Re6—Re4	90.092 (12)
Se1—Re2—Re6	118.490 (19)	Re3—Re6—Re4	59.875 (10)
Se5—Re2—Re6	58.400 (18)	Re3—Se1—Re2	63.085 (19)
Re3—Re2—Re6	60.089 (10)	Re3—Se1—Re1	63.381 (19)
P2—Re2—Re1	132.79 (5)	Re2—Se1—Re1	63.019 (19)
Se4—Re2—Re1	58.681 (17)	Re4—Se2—Re3	63.055 (17)
Se8—Re2—Re1	118.468 (19)	Re4—Se2—Re1	63.477 (17)
Se1—Re2—Re1	58.608 (17)	Re3—Se2—Re1	63.431 (18)
Se5—Re2—Re1	118.907 (18)	Re1—Se3—Re4	63.332 (17)
Re3—Re2—Re1	60.335 (11)	Re1—Se3—Re5	63.346 (18)
Re6—Re2—Re1	90.250 (12)	Re4—Se3—Re5	62.950 (16)
P2—Re2—Re5	137.53 (5)	Re2—Se4—Re5	63.646 (18)
Se4—Re2—Re5	58.314 (18)	Re2—Se4—Re1	63.170 (17)
Se8—Re2—Re5	58.284 (18)	Re5—Se4—Re1	63.452 (18)
Se1—Re2—Re5	118.757 (19)	Re6—Se5—Re2	63.029 (18)
Se5—Re2—Re5	117.90 (2)	Re6—Se5—Re3	63.085 (18)
Re3—Re2—Re5	89.848 (12)	Re2—Se5—Re3	62.925 (17)
Re6—Re2—Re5	59.510 (11)	Re3—Se6—Re6	63.300 (18)
Re1—Re2—Re5	60.184 (11)	Re3—Se6—Re4	63.097 (17)
P3—Re3—Se6	91.08 (5)	Re6—Se6—Re4	63.225 (17)
P3—Re3—Se1	92.48 (5)	Re6—Se7—Re4	63.279 (19)
Se6—Re3—Se1	176.42 (2)	Re6—Se7—Re5	62.771 (19)
P3—Re3—Se2	90.40 (5)	Re4—Se7—Re5	63.177 (19)
Se6—Re3—Se2	90.67 (2)	Re2—Se8—Re5	63.508 (18)
Se1—Re3—Se2	89.61 (2)	Re2—Se8—Re6	62.985 (18)
P3—Re3—Se5	93.57 (5)	Re5—Se8—Re6	62.651 (18)
Se6—Re3—Se5	89.43 (2)	O3—S1—O2	116.5 (4)
Se1—Re3—Se5	90.05 (2)	O3—S1—O1	109.9 (3)
Se2—Re3—Se5	176.03 (2)	O2—S1—O1	112.6 (3)
P3—Re3—Re2	136.60 (4)	O3—S1—C1	107.8 (4)
Se6—Re3—Re2	118.36 (2)	O2—S1—C1	107.2 (4)
Se1—Re3—Re2	58.503 (17)	O1—S1—C1	101.5 (4)
Se2—Re3—Re2	118.150 (18)	C11—P1—C15	104.1 (3)
Se5—Re3—Re2	58.489 (17)	C11—P1—C13	103.7 (4)
P3—Re3—Re4	133.15 (4)	C15—P1—C13	102.5 (4)
Se6—Re3—Re4	58.659 (17)	C11—P1—Re1	113.8 (2)
Se1—Re3—Re4	118.683 (19)	C15—P1—Re1	115.6 (3)
Se2—Re3—Re4	58.467 (18)	C13—P1—Re1	115.5 (2)
Se5—Re3—Re4	118.44 (2)	C23—P2—C21	104.3 (4)
Re2—Re3—Re4	90.236 (12)	C23—P2—C25	101.6 (4)
P3—Re3—Re6	135.68 (5)	C21—P2—C25	104.0 (4)
Se6—Re3—Re6	58.412 (18)	C23—P2—Re2	115.3 (3)
Se1—Re3—Re6	118.449 (19)	C21—P2—Re2	114.2 (3)
Se2—Re3—Re6	118.558 (19)	C25—P2—Re2	115.9 (3)
Se5—Re3—Re6	58.323 (18)	C33—P3—C31	104.8 (4)
Re2—Re3—Re6	59.958 (10)	C33—P3—C35	105.1 (4)
Re4—Re3—Re6	60.120 (11)	C31—P3—C35	101.1 (3)
P3—Re3—Re1	134.37 (5)	C33—P3—Re3	113.3 (2)
Se6—Re3—Re1	118.850 (19)	C31—P3—Re3	115.3 (3)
Se1—Re3—Re1	58.472 (17)	C35—P3—Re3	115.8 (2)
Se2—Re3—Re1	58.286 (16)	C43—P4—C45	100.0 (3)
Se5—Re3—Re1	118.346 (18)	C43—P4—C41	104.2 (4)
Re2—Re3—Re1	59.872 (10)	C45—P4—C41	104.4 (4)
Re4—Re3—Re1	60.217 (10)	C43—P4—Re4	115.6 (3)
Re6—Re3—Re1	89.907 (11)	C45—P4—Re4	117.0 (3)
P4—Re4—Se7	91.66 (5)	C41—P4—Re4	113.8 (3)
P4—Re4—Se2	92.20 (5)	C55—P5—C53	102.8 (5)
Se7—Re4—Se2	176.14 (2)	C55—P5—C51	105.0 (6)
P4—Re4—Se6	91.66 (4)	C53—P5—C51	98.4 (5)
Se7—Re4—Se6	89.39 (2)	C55—P5—Re5	115.9 (4)
Se2—Re4—Se6	90.42 (2)	C53—P5—Re5	116.1 (3)
P4—Re4—Se3	92.64 (4)	C51—P5—Re5	116.3 (3)
Se7—Re4—Se3	90.21 (2)	S1—O1—Re6	136.5 (3)
Se2—Re4—Se3	89.69 (2)	C6—C1—C2	118.0 (9)
Se6—Re4—Se3	175.69 (2)	C6—C1—S1	121.1 (7)
P4—Re4—Re3	134.62 (4)	C2—C1—S1	120.9 (8)
Se7—Re4—Re3	118.358 (19)	C1—C2—C3	121.2 (10)
Se2—Re4—Re3	58.478 (17)	C4—C3—C2	120.1 (10)
Se6—Re4—Re3	58.244 (18)	C3—C4—C5	118.9 (10)
Se3—Re4—Re3	118.399 (19)	C3—C4—C7	120.0 (9)
P4—Re4—Re5	135.26 (4)	C5—C4—C7	121.1 (11)
Se7—Re4—Re5	58.446 (17)	C4—C5—C6	120.1 (11)
Se2—Re4—Re5	118.418 (18)	C1—C6—C5	121.7 (10)
Se6—Re4—Re5	117.818 (19)	C12—C11—P1	116.8 (6)
Se3—Re4—Re5	58.526 (17)	C14—C13—P1	113.7 (5)
Re3—Re4—Re5	90.112 (12)	C16—C15—P1	115.6 (6)
P4—Re4—Re6	134.67 (4)	C22—C21—P2	117.0 (6)
Se7—Re4—Re6	58.355 (17)	C24—C23—P2	115.5 (5)
Se2—Re4—Re6	118.454 (18)	C26—C25—P2	115.4 (5)
Se6—Re4—Re6	58.243 (17)	C32—C31—P3	116.0 (6)
Se3—Re4—Re6	118.087 (18)	C34—C33—P3	115.7 (6)
Re3—Re4—Re6	60.005 (11)	C36—C35—P3	117.2 (5)
Re5—Re4—Re6	59.582 (11)	C42—C41—P4	116.5 (6)
P4—Re4—Re1	135.52 (4)	C44—C43—P4	116.2 (5)
Se7—Re4—Re1	118.598 (19)	C46—C45—P4	115.5 (5)
Se2—Re4—Re1	58.268 (17)	C52—C51—P5	117.6 (8)
Se6—Re4—Re1	118.376 (19)	C54—C53—P5	114.8 (7)
Se3—Re4—Re1	58.254 (17)	C56A—C55—P5	119.1 (12)
Re3—Re4—Re1	60.158 (11)	C56B—C55—P5	133.1 (18)
Re5—Re4—Re1	60.164 (10)	O4—S2—O6	112.0 (4)
Re6—Re4—Re1	89.807 (12)	O4—S2—O5	112.8 (4)
P5—Re5—Se4	94.95 (5)	O6—S2—O5	112.1 (5)
P5—Re5—Se7	89.06 (5)	O4—S2—C61	107.5 (4)
Se4—Re5—Se7	175.97 (2)	O6—S2—C61	105.4 (4)
P5—Re5—Se8	90.90 (5)	O5—S2—C61	106.5 (3)
Se4—Re5—Se8	88.59 (2)	C66—C61—C62	117.8 (7)
Se7—Re5—Se8	90.97 (2)	C66—C61—S2	122.5 (6)
P5—Re5—Se3	93.06 (5)	C62—C61—S2	119.7 (6)
Se4—Re5—Se3	90.01 (2)	C63—C62—C61	119.9 (8)
Se7—Re5—Se3	90.17 (2)	C62—C63—C64	122.4 (9)
Se8—Re5—Se3	175.91 (2)	C63—C64—C65	117.5 (8)
P5—Re5—Re6	132.11 (5)	C63—C64—C67	121.0 (9)
Se4—Re5—Re6	117.978 (19)	C65—C64—C67	121.5 (10)
Se7—Re5—Re6	58.574 (17)	C64—C65—C66	120.9 (9)
Se8—Re5—Re6	58.797 (17)	C61—C66—C65	121.6 (8)
Se3—Re5—Re6	118.763 (18)	Cl2S—C1S—Cl1S	114.6 (7)
P5—Re5—Re4	133.61 (5)	Cl3S—C2S—Cl4S	107.5 (8)
Se4—Re5—Re4	118.52 (2)	Cl6S—C2S—Cl5S	108.3 (8)

## References

[bb1] Beurskens, P. T., Beurskens, G., de Gelder, R., Smits, J. M. M., Garcia-Granda, S. & Gould, R. O. (2008). The *DIRDIF2008* program system. Crystallography Laboratory, Radboud University Nijmegen, The Netherlands.

[bb2] Bruker (2006). *SMART* and *SAINT*. Bruker AXS Inc. Madison, Wisconsin, USA.

[bb3] Dorson, F., Molard, Y., Cordier, S., Fabre, B., Efremova, O., Rondeau, D., Mironov, Y., Cîrcu, V., Naumov, N. & Perrin, C. (2009). *Dalton Trans.* pp. 1297–1299. 10.1039/b822105g 19462649

[bb4] Durham, J. L., Tirado, J. N., Knott, S. A., Oh, M. K., McDonald, R. & Szczepura, L. F. (2012). *Inorg. Chem.* **51**, 7825–7836. 10.1021/ic300877r 22765346

[bb5] Durham, J. L., Wilson, W. B., Huh, D. N., McDonald, R. & Szczepura, L. F. (2015). *Chem. Commun.* **51**, 10536–10538. 10.1039/c5cc03215f 26041404

[bb6] Endres, H. (1987). *Comprehensive Coordination Chemistry I*, edited by G. Wilkinson, p. 261. New York: Pergamon Press.

[bb7] Eremenko, I. L., Bakhmutov, V. I., Otl, F. & Berke, H. (1993). *Zh. Neorg. Khim.* **38**, 1653–1660.

[bb9] Knott, S. A., Templeton, J. N., Durham, J. L., Howard, A. M., McDonald, R. & Szczepura, L. F. (2013). *Dalton Trans.* **42**, 8132–8139. 10.1039/c3dt50436k 23584619

[bb10] Lindner, E. & Grimmer, R. (1971). *Chem. Ber.* **104**, 544–548.

[bb12] Mironov, Y. V., Brylev, K. A., Kim, S.-J., Kozlova, S. G., Kitamura, N. & Fedorov, V. E. (2011). *Inorg. Chim. Acta*, **370**, 363–368.

[bb13] Orto, P., Selby, H. D., Ferris, D., Maeyer, J. R. & Zheng, Z. (2007). *Inorg. Chem.* **46**, 4377–4379. 10.1021/ic7004985 17458960

[bb14] Sheldrick, G. M. (2008). *SADABS*. University of Göttingen, Germany.

[bb15] Sheldrick, G. M. (2015). *Acta Cryst.* C**71**, 3–8.

[bb16] Szczepura, L. F., Oh, M. K. & Knott, S. A. (2007). *Chem. Commun.* pp. 4617–4619. 10.1039/b708390d 17989810

[bb18] Yoshimura, T., Umakoshi, K., Sasaki, Y., Ishizaka, S., Kim, H.-B. & Kitamura, N. (2000). *Inorg. Chem.* **39**, 1765–1772. 10.1021/ic991282a 12526566

[bb19] Zheng, Z., Gray, T. G. & Holm, R. H. (1999). *Inorg. Chem.* **38**, 4888–4895. 10.1021/ic9906050 11671221

[bb20] Zheng, Z. & Holm, R. H. (1997). *Inorg. Chem.* **36**, 5173–5178.

