# Supplementary figures and images for: Crystal structure of octa-μ3-selenido-(p-toluene­sulfonato-κO)penta­kis­(tri­ethyl­phosphane-κP)-octa­hedro-hexa­rhenium(III) p-toluene­sulfonate di­chloro­methane disolvate
Source: Acta Crystallogr E Crystallogr Commun. 2015 Aug 6;71(Pt 9):m158–9. doi: 10.1107/S2056989015014334 (PMC4555408; doi:10.1107/S2056989015014334)

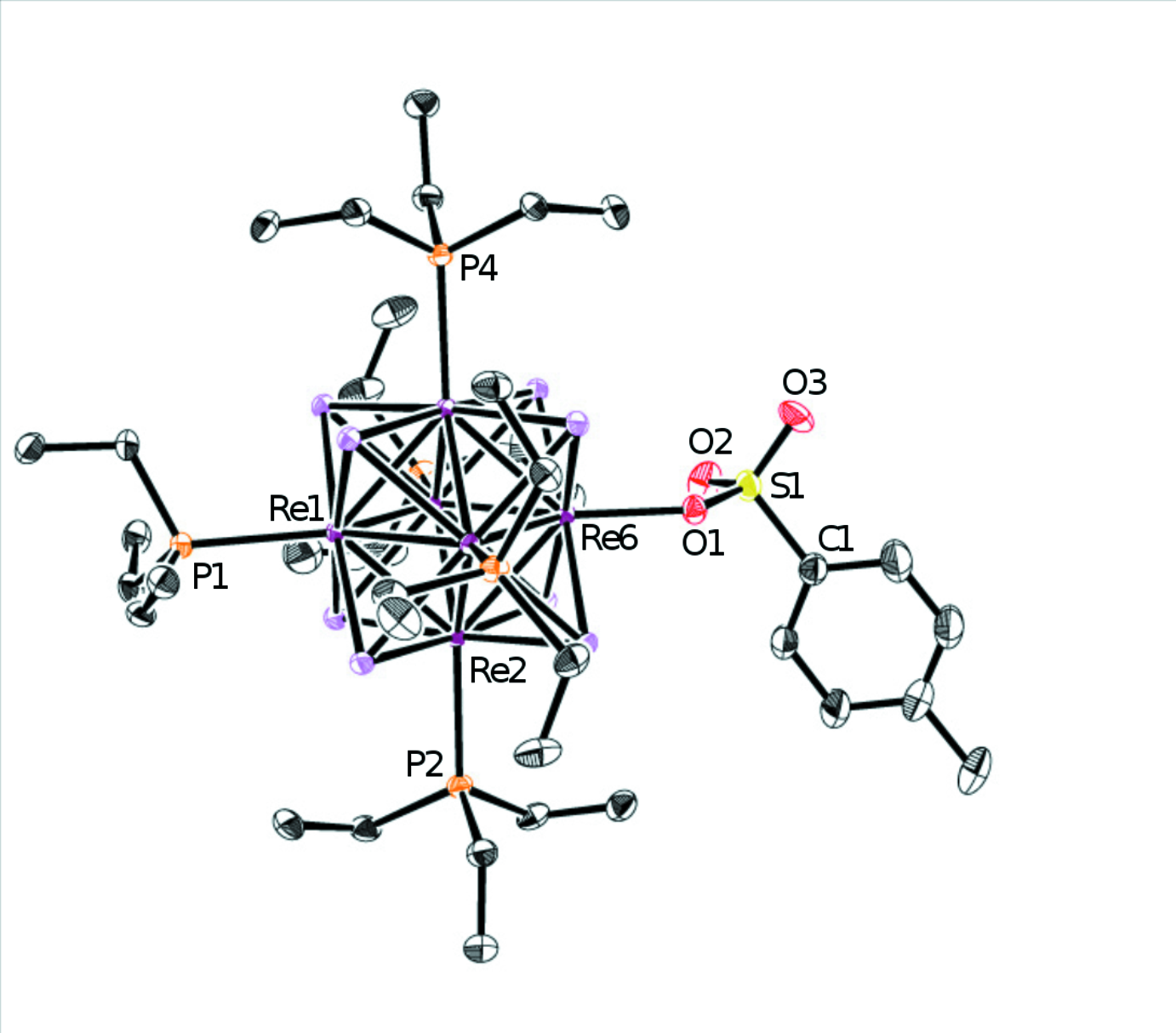

Supplement: Supplementary file 3 [file e-71-0m158-fig1.tif]
